# Dopamine suppresses octopamine signaling in *C. elegans*: possible involvement of dopamine in the regulation of lifespan

**DOI:** 10.18632/aging.100097

**Published:** 2009-10-21

**Authors:** Satoshi Suo, Joseph G. Culotti, Hubert H.M. Van Tol

**Affiliations:** ^1^ Samuel Lunenfeld Research Institute, Mount Sinai Hospital, Toronto, Ontario, Canada, M5G 1X5; ^2^ Department of Medical Genetics, University of Toronto, Toronto, Ontario, Canada, M5G 1X5; ^3^ Deceased, April 20, 2006.

**Keywords:** dopamine, octopamine, serotonin, food, lifespan, CREB

## Abstract

Amine neurotransmitters, such as dopamine, serotonin, and noradrenaline, play important roles in the modulation of behaviors and metabolism of animals. InC. elegans, it has been shown that serotonin and octopamine, an invertebrate equivalent of noradrenaline, also regulate lifespan through a mechanism related to food deprivation-mediated lifespan extension. We have shown recently that dopamine signaling, activated by the tactile perception of food, suppresses octopamine signaling and that the cessation of dopamine signaling in the absence of food leads to activation of octopamine signaling. Here, we discuss the apparent conservation of neural and molecular mechanisms for dopamine regulation of octopamine/noradrenaline signaling and a possible role for dopamine in lifespan regulation.

## Amine neurotransmitter regulation of life span

 It is becoming clear from studies in model animals that amine neurotransmitters can regulate the longevity of animals. In *Drosophila*, it is shown that a quantitative trait locus for the variation of longevity maps into the aromatic L-amino acid decarboxylase gene, which is required for dopamine and serotonin synthesis [1]. Murakami et al. showed that, in *C. elegans*, the serotonin receptor mutant *ser-1* has increased lifespan whereas another serotonin receptor mutant *ser-4* has decreased life span, suggesting that serotonin can affect lifespan in opposite ways depending on the receptor mechanism that is invoked [2]. It is also reported that serotonin signaling is required for reserpine-mediated lifespan extension [3].

Petrascheck et al. found through chemical screening that mianserin, an antidepressant, extends life span of *C. elegans* [4]. They demonstrated that mianserin is an antagonist for the serotonin receptor SER-4 and the octopamine receptor SER-3 and that mianserin-mediated lifespan extension was dependent on each of these receptors, suggesting that not only serotonin but also octopamine plays a role in the regulation of lifespan. Octopamine is an amine neurotransmitter that is considered to be a biological equivalent of noradrenaline [5]. It is shown that in *C. elegans* exogenous serotonin induces behavioral changes that are observed in the presence of food, whereas exogenous octopamine induces behaviors of starved animals [6]. It has been proposed therefore that serotonin and octopamine act as physiological antagonists and that serotonin signals the presence of food, whereas octopamine signals the absence of food. Thus, Petrascheck et al. tested the effect of mianserin under food deprivation since food deprivation has been shown to extend lifespan in many animals including *C. elegans* [7]. They found that mianserin did not further increase the lifespan of food-deprived animals, indicating that mianserin extends lifespan through aging mechanisms associated with food deprivation [4].

These results suggest that octopamine along with serotonin regulates lifespan in *C. elegans* through mechanisms that are related to food deprivation. We have recently elucidated a mechanism for activation of octopamine signaling in the absence of food in *C. elegans* and demonstrated the involvement of the amine neurotransmitter dopamine in this regulation [8,9]. We review the findings and discuss potential conservation with mammalian systems and a connection to aging.

## Octopamine signaling is activated in the absence of food

In *C. elegans*, activation of CREB can be detected using a *cre::gfp* fusion gene, in which the cyclic AMP response element (CRE) is fused to the gene encoding green fluorescent protein (GFP) [10]. In a strain carrying *cre::gfp*, GFP is expressed in cells in which CREB is activated. Using this reporter system, we first found that the absence of food induces CREB activation in the cholinergic SIA neurons [8]. To determine whether octopamine is involved in this signaling mechanism, mutants of the *tbh-1* gene were tested. *tbh-1 *encodes tyramine-ƒΐ-hydroxylase which is required for octopamine synthesis and is expressed only in the RIC neurons and the gonadal sheath cells (the latter are unlikely to play a role in this food response) [11]. *tbh-1* mutants failed to respond to the absence of food, indicating that octopamine is responsible for CREB activation. We also found that exogenous application of octopamine in the presence of food induces CREB activation in the SIA neurons, which also supports the involvement of octopamine. These results confirmed the notion that octopamine signaling is activated in the absence of food. Furthermore, we found that the octopamine receptor SER-3 is required for both responses to the absence of food and to exogenous octopamine. Cell-specific expression of SER-3 in the SIA neurons rescued exogenous octopamine and food responses of *ser-3* mutant animals, indicating that SER-3 works in the SIA neurons to receive octopamine signaling. Given that there is no synaptic connection between the RIC and SIA neurons, these results suggest that octopamine released from the RIC neurons humorally activates SER-3 in the SIA neurons in the absence of food.

## Dopamine suppresses octopamine signaling

Dopamine signaling in *C. elegans* is important for food sensing [12]. Dopaminergic neurons in *C. elegans* have sensory endings under the cuticle and sense the presence of food by mechanosensation [12-14]. The mechanosensation of food is believed to activate release of dopamine. Interestingly, one class of dopaminergic neurons, the CEP neurons, is known to be presynaptic to both the RIC and SIA neurons [13]. Considering that dopamine and octopamine are regulated oppositely by food and that dopaminergic neurons are in a suitable location to control octopamine signaling, we tested whether dopamine interacts with octopamine signaling [9].

We first found that exogenously applied dopamine suppresses exogenous octopamine-mediated CREB activation in the SIA neurons. To determine whether endogenous dopamine also suppresses octopamine signaling, we tested *cat-2* mutants, which are defective in dopamine synthesis since *cat-2* encodes the tyrosine hydroxylase, the rate limiting enzyme for dopamine synthesis [15]. *cat-2* mutants exhibited spontaneous CREB activation in the SIA neurons even in the presence of food. This spontaneous activation requires endogenous octopamine since spontaneous CREB activation was suppressed in *cat-2;tbh-1* double mutants. These results indicate that octopamine-SER-3-CREB signaling pathway is constitutively activated in *cat-2* mutants and dopamine normally suppresses this pathway in the presence of food.

To further demonstrate the involvement of endogenous dopamine in the suppression of octopamine signaling, we used the Sephadex beads. It was shown previously that the Sephadex beads induce a dopamine-dependent behavioral change presumably by mimicking the tactile attribute of food without providing nutritional or chemosensory cues associated with bacteria (food) [12]. Addition of the Sephadex beads to the culture plates completely suppressed CREB activation induced by the absence of food [9]. This result suggests that octopamine-mediated CREB activation in the absence of food is not initiated by the decrease in food intake (starvation) but by the absence of tactile perception of food by the dopaminergic neurons.

We have tested all identified dopamine receptors in *C. elegans* and found that two D2-like dopamine receptors, DOP-2 and DOP-3 [16,17], work downstream of dopamine to suppress octopamine signaling [9]. Cell-specific rescue experiments determined that both DOP-2 and DOP-3 work in the SIA neurons to suppress octopamine-mediated signaling. In addition, we found that DOP-3 also works in the RIC neurons to suppress CREB activation in response to endogenous dopamine. Therefore, it is likely that dopamine suppresses octopamine signaling in two ways. One is by affecting release of octopamine from the RIC neurons and the other is by negatively regulating the ability of octopamine to activate CREB in the SIA neurons.

**Figure 1.**
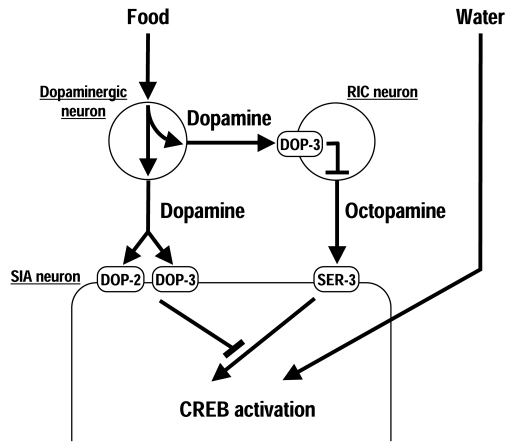
Regulation of CREB activation in the SIA neurons. In the presence of food, dopamine is released from the dopaminergic neurons and activates the dopamine receptor DOP-3 in the RIC neurons, possibly to decrease octopamine release. Dopamine also inhibits octopamine-mediated signaling in the SIA neurons through the dopamine receptors DOP-2 and DOP-3. In the absence of food, cessation of dopamine signaling results in octopamine-mediated CREB activation through the octopamine receptor SER-3. Exposure to water also induces CREB activation in the SIA neurons independently of dopamine and octopamine.

These studies suggests that*C. elegans* uses a three-neuron-type circuit to control octopamine signaling in response to food (Figure 1). In the presence of food, dopamine is released by the dopaminergic neurons. The released dopamine activates DOP-3 in the RIC neurons, possibly to decrease octopamine release. Simultaneous-ly, dopamine also inhibits octopamine-mediated signaling in the SIA neurons through DOP-2 and DOP-3. In the absence of food, dopamine is not released, which inactivates DOP-3 in the RIC neurons, potentially increasing octopamine release. The released octopamine activates the octopamine receptor SER-3 in the SIA neurons, which results in activation of CREB because negative regulation by dopamine receptors does not occur when dopamine is not released. An important feature of this circuit is that octopamine signaling can be activated solely by removal of suppression by dopamine signaling without any other signaling to activate it.

There are striking analogies between this three-neuron-type circuit in *C. elegans* and a three-neuron-type circuit possibly involved in food response in the mammalian brain. First, food stimuli increase dopamine and decrease noradrenaline release in the mammalian brain [18,19]. Second, noradrenergic neurons in the locus coeruleus receive projections from dopaminergic neurons in the ventral tegmental area [20] and their firing rate is negatively regulated by dopamine [21]. Third, both the noradrenergic neurons and the dopaminergic neurons innervate basal forebrain cholinergic neurons [22]. This may be analogous to the way the octopaminergic RIC neurons and dopaminergic neurons (e.g. the CEP neurons) signals to the cholinergic SIA neurons in *C. elegans*. Therefore, we postulate that the neuronal and molecular circuitry for food sensing we have discovered in *C. elegans* is conserved in vertebrates, in which cessation of dopamine signaling activates octopamine/noradrenaline signaling.

## Potential role of dopamine in the lifespan regulation

It is unknown whether the SIA neurons play any role in aging and it is highly possible that mianserin-mediated lifespan extension work through its effect on SER-3 in other cells. However, the finding that dopamine regulates the octopaminergic RIC neurons suggests that octopamine signaling in the cells other than the SIA neurons are also regulated by dopamine. This raises the possibility that dopamine plays a role in the food-mediated regulation of lifespan.

It has been suggested that food limits lifespan through at least two different mechanisms. One is by providing nutrition and the other is by providing sensory perception [23]. Since dopamine regulates octopamine signaling in response to tactile perception of food rather than ingestion of food, if dopamine plays a role in lifespan regulation, it would be because of its involvement in food perception.

Murakami et al. showed that dopamine-deficient *cat-2* mutants have a normal lifespan when measured in standard culture conditions [2]. However, this result does not rule out the possible involvement of dopamine since molecular mechanisms that control lifespan are highly context dependent [24]. In fact, mianserin-mediate lifespan extension is not observed in the standard culture condition in which animals are grown on solid agar but it is observed only in a liquid culture [25,26]. Intriguingly, soaking animals in water also induces CREB activation in the SIA neurons just as is seen in the absence of food (Figure 1) [8], suggesting the possible existence of an interaction between food signaling and signaling mediated by the exposure to liquid. Therefore, more detailed studies of the effect of dopamine signaling on the regulation of lifespan in *C. elegans* would be particularly enlightening, especially since it has been reported that a polymorphism in the tyrosine hydroxylase gene, which is required for dopamine synthesis, is associated with variation in human longevity [27,28].

## Acknowledgement

This work was supported in part by the Canadian Institutes of Health Research grant MOP-77722 and MOP-82909 to J.G.C. J.G.C. and H.H.M.V.T. are holders of Canadian Research Chairs. S.S. is a recipient of a Parkinson Society Canada Basic Research Fellowship.
